# Length of Stay in Hospice Care across Racial/Ethnic Minorities over 65 Years of Age: A Descriptive Analysis

**DOI:** 10.1093/geroni/igab046.2854

**Published:** 2021-12-17

**Authors:** Heshuo Yu, J Scott Brown

**Affiliations:** 1 Miami University in Oxford, Oxford, Ohio, United States; 2 Miami University, Oxford, Ohio, United States

## Abstract

**Purpose:**

This study aims to explore the relationship between race/ethnicity and length of stay in hospice care among adults over 65 years of age in the United States. This topic is understudied within a population-representative sample, particularly among non-White decedents.

**Methods:**

Secondary analysis of data from the 2007 NHHCS (n=3,918). Race/ethnicity included Hispanics/Latinos, Non-Hispanic Whites, African Americans, and other races. Length of hospice stay was measured by the number of days that patients received hospice care from hospice agencies.

**Results:**

The study found that African Americans have a longer length of stay in hospice agencies than Whites, even after controlling for all other factors in the model. Female gender, older age, and several diseases are covariates that significantly impact length of hospice stay.

**Discussion:**

Compared to other races/ethnicities, the long length of stay in hospice among African Americans may negatively impact the quality of end-of-life care and quantity of skilled staff visits. Future research is recommended to further explore potential consequences of longer hospice stays, especially within African American communities. Studies with larger samples of minorities that integrate socioeconomic factors need to be done to better study the relationship between length of hospice stay and race/ethnicity.

